# Modifying oncolytic virotherapy to overcome the barrier of the hypoxic tumor microenvironment. Where do we stand?

**DOI:** 10.1186/s12935-022-02774-w

**Published:** 2022-11-24

**Authors:** Sara Shayan, Arash Arashkia, Kayhan Azadmanesh

**Affiliations:** Department of Molecular Virology, Pasteur Institute of Iran, No. 69, Pasteur Ave, Tehran, Iran

**Keywords:** Oncolytic virus, Hypoxia, Normoxia, Tumor microenvironment

## Abstract

Viruses are completely dependent on host cell machinery for their reproduction. As a result, factors that influence the state of cells, such as signaling pathways and gene expression, could determine the outcome of viral pathogenicity. One of the important factors influencing cells or the outcome of viral infection is the level of oxygen. Recently, oncolytic virotherapy has attracted attention as a promising approach to improving cancer treatment. However, it was shown that tumor cells are mostly less oxygenated compared with their normal counterparts, which might affect the outcome of oncolytic virotherapy. Therefore, knowing how oncolytic viruses could cope with stressful environments, particularly hypoxic environments, might be essential for improving oncolytic virotherapy.

## Background

**Fig. 1:**
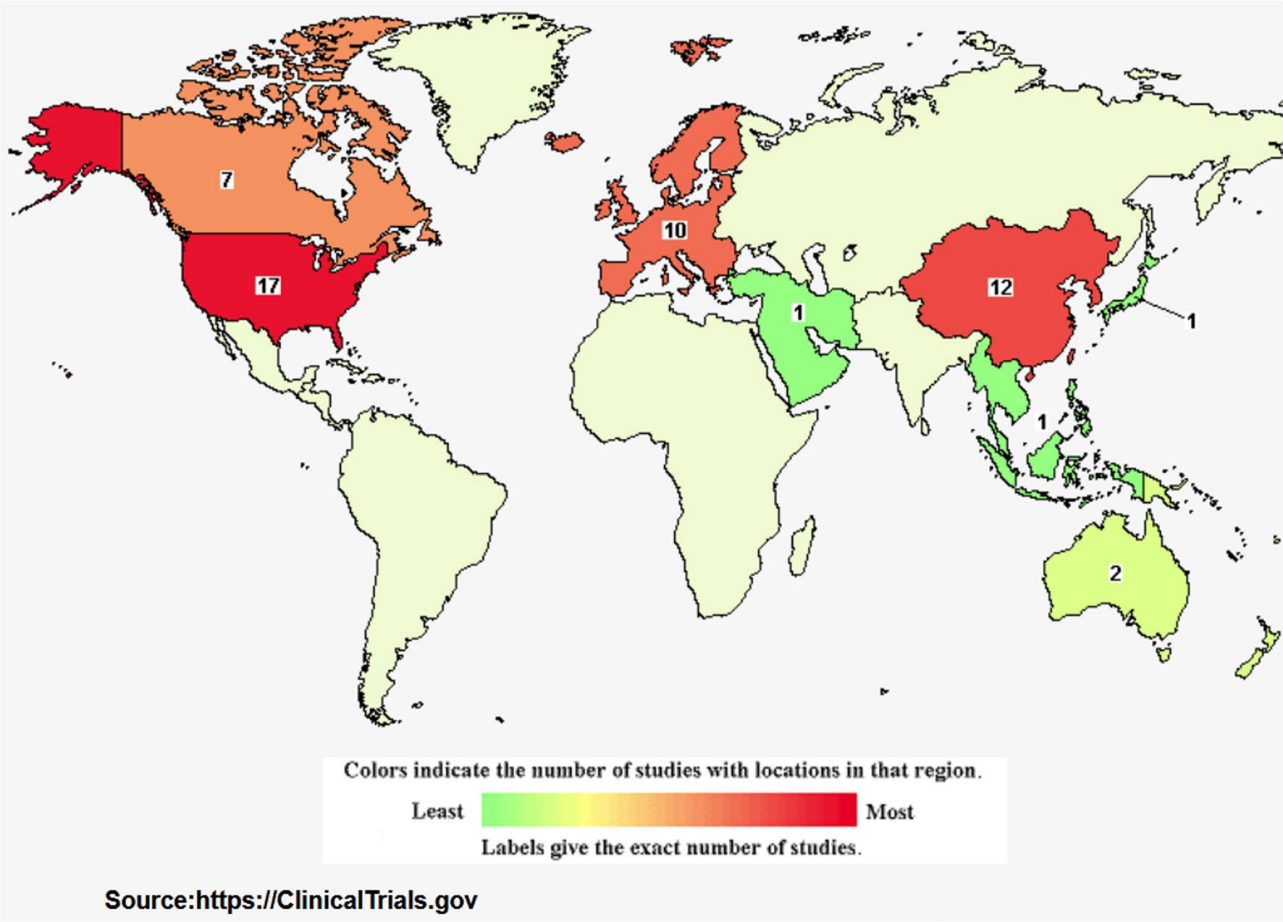
The map indicates the number of oncolytic virotherapy studies (ongoing or completed phase 2 or 3 clinical trials) based on region. Source https://clinicaltrials.gov/ Jun 2022

The use of oncolytic viruses (OVs) for cancer therapy has become a promising approach in recent years. Some viruses are naturally capable of killing cancer cells [1]. However, some OVs need to be modified in a way that allows them to selectively replicate in cancer cells without harming normal tissues [2–5]. Such viruses can also be used as delivery vehicles by harboring the gene of interest [2]. The idea of using viruses to treat cancer cells has revolutionized the traditional cancer therapies. Currently, many OVs have been evaluated in clinical trials for treating different types of cancers (Table 1, Fig. 1). Yet, only four OVs have been approved for cancer therapy. In 2004, RIGVIR, as an unmodified enterovirus, was approved in Latvia as the first OV for the treatment of melanoma [6]. However, the approval was withdrawn in 2019 [6]. Additionally, Oncorine, an engineered adenovirus H101, was approved in China in 2005 for treating head and neck cancer [7]. In 2015, for the first time, the US Food and Drug Administration (FDA) approved an oncolytic herpes simplex virus-1 (HSV-1) known as IMLYGIC (Talimogene laherparepvec), developed by BioVex, for the treatment of melanoma [8]. In 2021, Delytact, a genetically engineered oncolytic herpes simplex virus type 1 (G47Δ /oHSV-1), has received conditional approval from Japan’s Ministry of Health, Labor and Welfare (MHLW) as an oncolytic virotherapy for the treatment of patients with malignant glioma in Japan [9]. There is growing evidence that the success of oncolytic virotherapy depends on the tumor microenvironment (TME) (Table 2). Hypoxia is the hallmark of TME, which might present an obstacle to the effectiveness of OV therapy [10]. Therefore, understanding the impact of low oxygen tension on OVs is essential to improve the anti-tumor effect of OV therapy. In this review, characteristics of the hypoxic microenvironment and different types of modifications that have been applied to improve the efficacy of OVs during hypoxia have been summarized.

**Table 1:** The table indicates the ongoing or completed phase 2/3 clinical trials of OVs. Clinical Trials search filters: “Recruitment: Recruiting and completed. Study type: Interventional. Study result: All. Study Phase:2/3/4.” Source: https://clinicaltrials.gov/. Jun 2022

Study title	Intervention	Condition	Phase	Clinicaltrials.gov Identifier
Intraperitoneal injection of oncolytic viruses H101 for patients with refractory malignant ascites	Drug: oncorine (H101)	Refractory malignant ascites	2	NCT04771676
OH2 oncolytic viral therapy in central nervous system tumors	Biological: OH2 injection	Central nervous system tumors	2	NCT05235074
Safety and efficacy of CG0070 oncolytic virus regimen for high grade NMIBC after BCG failure	Biological: CG0070	Bladder cancer	2	NCT02365818
A study of combination with TBI-1401(HF10) and ipilimumab in Japanese patients with unresectable or metastatic melanoma	Biological: TBI-1401(HF10) drug: ipilimumab	Melanoma stage III melanoma stage IV	2	NCT03153085
A study of combination treatment with HF10 and ipilimumab in patients with unresectable or metastatic melanoma	Biological: HF10 plus ipilimumab	Malignant melanoma	2	NCT02272855
INCMGA00012 and pelareorep for the treatment of metastatic triple negative breast cancer, IRENE study	Biological: pelareorep	Anatomic stage IV breast cancer AJCC v8	2	NCT04445844
LOAd703 oncolytic virus therapy for pancreatic cancer	Genetic: delolimogene mupadenorepvec drug:gemcitabine drug:nabpaclitaxel biological:atezolizumab	Pancreatic cancer	2	NCT02705196
OH2 oncolytic viral therapy in pancreatic cancer	Biological:OH2 injection (OHSV-2)	Pancreatic cancer	2	NCT04637698
OH2 oncolytic viral therapy in solid tumors	Biological:OH2 injection, with or without irinotecan or HX008	Solid tumor gastrointestinal cancer	2	NCT03866525
OH2 injection in combination with HX008 for melanoma	Biological: OH2 injection, HX008 injection	Melanoma	2	NCT04616443
OH2 injection in solid tumors	Biological: OH2 injection Drug: Keytruda	Solid tumor melanoma	2	NCT04386967
A study of recombinant vaccinia virus to treat malignant melanoma	Biological: JX-594	Melanoma	2	NCT00429312
Parvovirus H-1 (ParvOryx) in patients with progressive primary or recurrent glioblastoma multiform	Drug: H-1PV	Glioblastoma multiform	2	NCT01301430
Safety and efficacy study of REOLYSIN® in the treatment of bone and soft tissue sarcomas metastatic to the lung	Biological: REOLYSIN®	Osteosarcoma ewing sarcoma family tumors malignant fibrous histiocytoma	2	NCT00503295
A study of talimogene laherparepvec in stage IIIc and stage IV malignant melanoma	Drug: talimogene laherparepvec	Melanoma	2	NCT00289016
Study evaluating cemiplimab alone and combined with RP1 in treating advanced squamous skin cancer	Drug: cemiplimab Biological: RP1	Cutaneous squamous cell carcinoma advanced cutaneous squamous cell carcinoma metastatic cutaneous squamous cell carcinoma	2	NCT04050436
Study of RP1 monotherapy and RP1 in combination with nivolumab	Biological: RP1 Biological: nivolumab	Cancer melanoma (skin) mismatch repair deficiency	2	NCT03767348
A Study of GL-ONC1, an oncolytic vaccinia virus, in patients with advanced peritoneal carcinomatosis	Biological: GL-ONC1	Peritoneal carcinomatosis	2	NCT01443260
A study to assess overall response rate by inducing an inflammatory phenotype in metastatic BReast cAnCEr with the oncolytic reovirus PeLareorEp in combination with anti-PD-L1 avelumab and paclitaxel—BRACELET-1 study	Drug: paclitaxel Biological: pelareorep Drug: avelumab	Breast cancer metastatic	2	NCT04215146
A phase 2b study of modified vaccinia virus to treat patients advanced liver cancer who failed sorafenib	Biological: JX-594 Recombinant Vaccina GM-CSF other: best supportive car	Hepatocellular carcinoma liver cancer HCC	2	NCT01387555
A study of recombinant vaccinia virus to treat unresectable primary hepatocellular carcinoma	Genetic: JX-594: recombinant vaccinia virus (TK-deletion plus GM-CSF)	Carcinoma, hepatocellular	2	NCT00554372
Hepatocellular carcinoma study comparing vaccinia virus based immunotherapy plus sorafenib vs sorafenib alone	Biological: pexastimogene devacirepvec (Pexa Vec) Drug: sorafenib	Hepatocellular carcinoma (HCC)	2	NCT02562755
Phase 2 study of REOLYSIN® in combination with paclitaxel and carboplatin for non-small cell lung cancer with KRAS or EGFR activation	Biological: REOLYSIN® drug: carboplatin drug: paclitaxel	Carcinoma, non-small cell lung	2	NCT00861627
Study of TBio-6517 Given alone or in combination with pembrolizumab in solid tumors	Biological: TBio-6517 biological: pembrolizumab	Solid tumor microsatellite stable colorectal cancer HPV positive oropharyngeal squamous cell carcinoma	2	NCT04301011
Intrapleural administration of HSV1716 to treat patients with malignant pleural mesothelioma	Biological: HSV1716 intra-pleural delivery	Malignant pleural mesothelioma	2	NCT01721018
Oncolytic MG1-MAGEA3 with Ad-MAGEA3 vaccine in combination with pembrolizumab for non-small cell lung cancer patients	Biological: BT-001 biological: pembrolizumab [Keytruda]	Solid tumor, adult metastatic cancer soft tissue sarcoma	2	NCT02879760
A clinical trial assessing BT-001 alone and in combination with pembrolizumab in metastatic or advanced solid tumors	Biological: BT-001 biological: pembrolizumab [keytruda]	Solid tumor, adult metastatic cancer soft tissue sarcoma	2	NCT04725331
Recombinant vaccinia virus administered intravenously in patients with metastatic, refractory colorectal carcinoma	Biological: JX-594 drug: irinotecan	Colorectal carcinoma CRC	2	NCT01394939
UARK 2014–21 a phase II Trial of oncolytic virotherapy by systemic administration of edmonston strain of measles virus	Drug: MV-NIS	Multiple myeloma	2	NCT02192775
Safety, tolerability and pharmacokinetics characteristics of recombinant oncolytic vaccinia virus injection t601 as a single drug or in combination with oral flucytosine (5-FC), in patients with advanced malignant solid tumors	Biological: T601 combination product: T601 + 5-FC	Advanced malignant solid tumors	2	NCT04226066

**Table 2:** List of 10 most relevant literature review articles

Author and reference number	Year of publication	Title	Journal
Yun Shin Chun [11]	2005	Employing tumor hypoxia for oncolytic therapy in breast cancer	Journal of Mammary Gland Biology and Neoplasia
Hay JG [12]	2005	The potential impact of hypoxia on the success of oncolytic virotherapy	Current Opinion in Molecular Therapeutics
Jayson Hardcastle [13]	2007	Oncolytic viruses driven by tumor-specific promoters	Curr Cancer Drug Targets
Han Hsi Wong [14]	2010	Oncolytic viruses for cancer therapy: overcoming the obstacles	Viruses
Jeffrey Wojton [15]	2010	Impact of tumor microenvironment on oncolytic viral therapy	Cytokine & Growth Factor Reviews
Sheng Guo, Z [10]	2011	The impact of hypoxia on oncolytic virotherapy	Virus Adaptation and Treatment
Hiroshi Fukuhara [16]	2016	Oncolytic virus therapy: a new era of cancer treatment at dawn	Cancer science
Carole Achard [17]	2018	Lighting a fire in the tumor microenvironment using oncolytic immunotherapy	EBiomedicine
Agata Hadryś [18]	2020	Mesenchymal stem cells as carriers for systemic delivery of oncolytic viruses	Eur J Pharmacol
Wang L [19]	2022	Remodeling the tumor microenvironment by oncolytic viruses: beyond oncolysis of tumor cells for cancer treatment	Journal for ImmunoTherapy of Cancer

### Hypoxic tumor microenvironment and related barriers to conventional cancer therapy

The tumor microenvironment (TME) is the ecosystem around tumor or cancer stem cells [20]. A growing number of studies have emphasized the importance of TME in recurrence, metastasis, and the development of drug resistance by cancer cells [21, 22]. TME comprises blood vessels, lymph vessels, immune cells, and proliferating tissue [23]. One of the major features of the TME in solid tumors is hypoxia, which has been defined as a state in which adequate oxygen is not available. Based on previous studies, there are two types of hypoxic conditions. First, diffusion-limited hypoxia or chronic hypoxia leads to inhibition of cell proliferation in cancer regions with low oxygen concentration. In chronic hypoxia, oxygen poorly diffuses throughout the tissues, and therefore, cells within this limiting distance, approximately 100 µm, do not have access to oxygen. In other words, the demand for oxygen in tumor cells exceeds the supply, and as a result, more blood vessels are formed. Nevertheless, these aberrant blood networks fail to meet the cells’ oxygen requirement, and eventually, the oxygen level drops to 1–2% as hypoxia is induced [24, 25]. The other form is acute hypoxia or perfusion-limited hypoxia, which is a temporary interruption in oxygen perfusion or fluctuation in oxygen level for several minutes [25]. These hypoxic regions undergo cell death and create necrotic zones [26]. Different aspects of tumor biology are affected by hypoxia, such as metabolism, cell signaling, and modification of RNA and DNA [27]. It was shown that cancer cells adapting to hypoxia display a more aggressive phenotype and become more resistant to therapeutic strategies [28]. A number of studies have shown that hypoxia may confer resistance directly or indirectly to conventional therapy, including radio-chemotherapy [29, 30]. Additionally, cancer stem cells located in the hypoxic niche of tumors involved in epithelia-mesenchymal transition (EMT) are naturally resistant to chemotherapy or radiotherapy [31]. Radiotherapy demands sufficient oxygen to exert a cytotoxic effect on tumor cells; therefore, hypoxia could directly induce resistance to radiotherapy [32]. Some therapeutic agents, including cyclophosphamide, carboplatin, and doxorubicin, require oxygen to eradicate cancer cells. But reduced blood flow in acute hypoxia and greater diffusion distances, which are seen in chronic hypoxia, affect the distribution of such chemotherapeutic agents [28]. Hypoxia can also indirectly decrease the effect of cancer therapies by regulating post-transcriptional and transcriptional gene expression (2). Such alterations in gene expression due to decreased oxygenation may lead to increased invasiveness, angiogenesis, metastasis, and drug resistance, all of which may reduce the efficacy of chemotherapy [33].

### Barriers to oncolytic virotherapy during hypoxia and recent advances

The hypoxic microenvironment is a major limitation that should be tackled to improve the efficacy of virotherapy [15]. There are crucial factors that should be considered when designing an OV to eradicate cancer cells under hypoxic conditions, including viral delivery and distribution, the immunosuppressive tumor microenvironment, and viral replication [10].

### Viral delivery and distribution

One of the major challenges during oxygen tension is the optimal delivery and spread of the OVs. In general, there are two strategies for administration of OVs, including intravenous and intratumoral delivery [34]. OVs can be delivered intratumorally, which is the most common method for cancer therapy in preclinical or clinical trials. However, this approach may not be useful for patients with multicentric or metastatic cancers [35–37]. OVs can also be delivered systemically to treat metastatic diseases. Recent studies indicate that immune effector cells, including T and NK cells, are dysfunctional in hypoxic zones [38]. But following OVs injection, they undergo multiple cycles of replication and are able to induce substantial numbers of immune cells to enter tumors [39]. Notably, during hypoxia, vascular endothelial growth factor (VEGF) expression is upregulated [40], leading to vascular permeability, which also facilitates the infiltration of immune cells. The increased immune cell infiltration into the tumor environment might result in viral clearance and reduce the effectiveness of oncolytic virotherapy [41]. Therefore, it is needed to mitigate the early immune responses to allow OV replication and spread throughout the tumor [39].

One approach to addressing these challenges is using OVs that naturally reduce VEGF expression. Hou et al. showed that the vaccinia virus possesses an antiangiogenic ability that suppresses VEGF expression during active oncolytic vaccinia infection, yet the mechanism of action is unknown [42]. They proposed that combination therapy with antiangiogenic agents could help extend the effectiveness of the treatment after clearance of the oncolytic vaccinia virus [42]. Carew et al. indicated that Reolysin (unmodified human Reovirus) could down-regulate HIF-1, HIF-2, and VEGF expression (43). Of note, hypoxia-inducible factors (HIFs) are key molecules that regulate cellular responses to hypoxia. HIF has been shown to be one of the modulators of host cell innate immune response to virus infection by overexpression of interferons (IFNs) and other gene transcripts with anti-viral activity [44]. Hence, down-regulation of HIF can improve oncolytic therapy against various types of tumors.

An alternative approach to systemic delivery of OVs is using other cells as a vehicle. Several studies have shown that these cells support viral replication; therefore, making them attractive candidates for OVs delivery. In fact, these cells are loaded with OVs and shield them from immune-mediated neutralization during migration to tumor sites [45]. After reaching tumor cells, OVs replicate within the tumor cells but may not reach the hypoxic core. Thus, using cells as delivery vehicles that target the hypoxic region of tumor cells may improve OV therapy [46].

Tumor hypoxia in glioblastoma (GBM) is an important factor in tumor aggression and progression, but targeting the hypoxic region of glioma remains a significant therapeutic challenge. Glioblastoma originates from neural stem cells (NSCs) [46] and it has been demonstrated that hypoxia is a critical factor in NSC glioma tropism. Furthermore, it was indicated that NSC is one of the potential virus carriers capable of supporting the replication and release of adenovirus progeny to glioma cells. Therefore, NSC loaded with adenovirus can migrate to the hypoxic area of GBM and allow the virus to spread throughout the tumor. Currently, the immortalized NSC line is approved by the FDA for clinical trials [46]. In the glioma model, MSCs are another possible carrier for adenovirus delivery [47]. It was indicated that hypoxia-induced VEGF could induce the homing of MSC to tumor sites in murine glioma. Following systematic injection of MSC-Ad35, they were detectable in tumors and also spared in the non-tumoral areas. It was shown that MSC-Ad35 could reduce tumor growth, but Ad35 alone could not even reach the tumor site. The other approach is modifying the genomes of viruses [47]. Yousaf et al. systematically administered an oncolytic adenovirus type 5, harboring a firefly luciferase gene under the control of the major-late promoter (EnAd), against colorectal solid tumor xenografts. They indicated that the protein expression of HIF-1 was decreased during the late phase of the viral life cycle, resulting in the down-regulation of angiogenic factors, including VEGF [48]. In addition, to maximize the distribution and killing capability of oncolytic adenovirus in renal cancer cells under hypoxic conditions, Zhang et al. generated an oncolytic adenovirus expressing Decorin while carrying the Ki67 promoter upstream of the hypoxia response element (HRE). Their results demonstrated that Decorin reduced collagen fibers and improved the spread of the viruses within tumor cells, and HRE-Ki67-Decorin had a higher ability to suppress tumor growth under hypoxic conditions than Ad-Decorin [49].

Another approach, apart from modifying the OV itself, is combination therapy. Kurozumi et al. reported that antiangiogenic treatment with cyclic RGD (cRGD) peptide before oHSV-1 (ICP34.5- and ICP6-) therapy reduced the viral clearance and increased the oHSV-1 efficacy in glioma tumor cells [50]. In another study, Matuszewska et al. hypothesized that intravenous delivery of oncolytic NDV may result in vascular shutdown (a phenomenon that slows down tumor growth) and increased tumor hypoxia. Therefore, they used 3TSR (Thrombospondin-1 three-type 1 repeat, collectively referred to as 3TSR) to induce tumor regression and normalize tumor vasculature before administration of oncolytic NDV. They indicated that combined 3TSR therapy with oncolytic NDV (F3aa) improved leukocytes’ infiltration into the ovarian tumors. They also showed that 3TSR treatment decreased the percentage of hypoxic tumor cells, improved tumor perfusion that led to increased OV efficacy, and provided a favorable environment for immune cell infiltration. In vivo results indicated that the combination of 3TSR and NDV resulted in tumor growth inhibition [51].

### Immunosuppressive tumor microenvironment

The hypoxic tumor microenvironment suppresses the immune cells, which poses a great challenge to cancer immunotherapy [52]. Yet, the immunosuppressive TME is favorable for viral infection. For instance, anti-viral responses of interferons, which are the first-line of defense against viral infection, are impaired during hypoxia, by which cancer cells can escape immune evasion [53]. Moreover, IFN-regulated genes induce the transcription of major histocompatibility complex (MHC) class I antigen presentation. However, hypoxia leads to downregulation of MHC, which is linked to the reduced T cell infiltration into tumors [54]. It was indicated that impaired IFN signaling in various tumor cells would allow the tumor-specific replication of the OVs. But Kurokawa et al. showed that cancer cells can have intact IFN signaling to regulate virus replication. Therefore, the anti-viral elements might serve as biomarkers to help improve therapeutic outcomes by identifying individual cancer patients who are most likely to benefit from OV treatment [55].

Another critical barrier for OVs is the tumor cells that can escape immune surveillance. It is true that OVs have the ability to convert cold tumors to hot tumors, but cancer cells can also adapt to hypoxia by overexpression of hypoxia-inducible factors (HIFs) [56, 57]. Recent studies have shown that HIF downregulates the expression of MHC on tumor cells; thereby, cancer cells can escape from T cells recognition [54]. In view of this, bi-specific T cell engagers (BiTEs) are a promising approach to circumvent this challenge [58], although there are hurdles to be overcome in using BiTEs. First, there is a toxicity associated with the systemic administration of many BiTEs. In addition, BiTEs need to penetrate the TME to induce the killing of tumor cells [59]. To overcome these challenges, OVs have been modified to encode BiTEs. Of note, one of the ideal tumor-associated antigens (TAA) that is expressed during hypoxia is the epithelial cell adhesion molecule (EpCAM). Accordingly, Friedman et al. demonstrated that EpCAM-CD3 BiTE encoding in adenovirus could activate T cells and eradicate cancer cells more efficiently [60–62].

Recent works have shown a crosstalk between TME and cancer-associated fibroblasts (CAFs) [63]. CAFs have a valuable role in tumor progression, making them a valuable target for transforming the TME. Therefore, OVs, including vaccinia virus and adenovirus, have been modified to express fibroblast-activating protein CD3 BiTE to transform the immunosuppressive TME, which plays an important role in the development and survival of cancer cells [64].

Another strategy to improve the therapeutic efficacy of the OVs within the immunosuppressive TME of cancer cells is the coadministration of an OV with chimeric-antigen receptor (CAR) T cells. The CAR T cell is one of the recent advances in cancer treatment. In fact, T cells are engineered to express artificial T cell receptors to recognize cancer cells more efficiently. However, tumor cells evade immune cells by losing the expression of the target antigen [65]. One strategy to overcome this obstacle is using an OV that expresses CAR-targeted TAA [66]. Recently, an adenovirus has been modified to express EGFR-targeting BiTE in combination with CAR T cells. The results showed that the combination of OAD-BiTE with CAR T cells significantly improved the activation and infiltration of T cells [66].

### Viral replication

Another barrier that may need to be overcome is the capability of OVs to replicate within tumor cells under hypoxic conditions. During cellular stresses such as hypoxia and viral infection, cellular protein synthesis is reduced, helping the cells to overcome the unfavorable situation [67]. Since viruses are dependent on the host protein synthesis machinery to synthesize their own proteins, protein shut down during hypoxia could largely affect viral replication and the efficacy of oncolytic virotherapy [68]. Some OVs have an inherent capacity to replicate during hypoxia, including NDV, VACV, VSV, and RV. Others, such as HSV-1 and Ad, must be modified to improve their replication in low oxygen environments. The following section provides key findings from the studies regarding the replication of OVs in the setting of hypoxic tumors.

### Herpes simplex virus type 1 (HSV-1)

Several studies have shown the impact of hypoxia on oncolytic herpes simplex virus type 1 (oHSV-1). Aghi et al. reported that hypoxic microenvironments boosted the replication of HSV G207, in which both copies of ICP34.5 and UL39 were deleted. Based on their study, HSV G207 could replicate more efficiently during hypoxia compared to normoxia due to the increased expression of GADD34 in hypoxic U87 GBM cells. Of note, UL39 encodes the ribonucleotide reductase (RR), which is responsible for converting ribonucleotide to deoxyribonucleotide. Rapidly dividing cancer cells produce an adequate amount of RR; therefore, HSV G207 can replicate in and lyse these cancer cell types [69]. However, hypoxia blocks the S phase of the cell cycle, which may reduce viral replication in cancer cells [70]. Accordingly, Reinblatt et al. combined HSV G207 with a multimerized hypoxia-responsive enhancer (10xHRE) to improve the cytotoxicity of HSV G207 under hypoxic conditions in CT26 cancer cells. Their results showed that HSV G207 cytotoxicity increased in CT26 cells transfected with 10xHRE-UL39 during hypoxia, while there was no improvement in normoxic conditions [70].

In another study, Fasullo et al. demonstrated that during hypoxia, MDA-MB-231 (P53-/-) cells were more permissive to HSV-1 derived R3616, in which both copies of ICP34.5 were deleted, and a LacZ–coding sequence was inserted in the UL39 locus, compared to MCF-7 (P53 +) cells. Their results indicated that the titer of R3616 collected from MDA-MB-231 was high, and they hypothesized that hypoxia could improve replication of the OV [71]. In our previous study, we demonstrated that HSV-HMGB1 (lacking both copies of ICP34.5 and harboring the HMGB1 sequence in the TK locus) could kill colorectal cancer cells more efficiently than the parental virus during hypoxia or normoxia, except for the HT29 cell line, in which HSV-HMGB1 enhanced the viability of cells under hypoxic conditions. Moreover, we showed that HSV-HMGB1 induced autophagy in HT29 cells during hypoxia [72].

Another oHSV-1 known as G47Δ (ICP34.5- ICP6- LacZ + ICP47-), was examined by Sgubin et al. in glioblastoma stem cells (GSCs) during normoxia and hypoxia. Studies have illustrated that CD133 expression increases in human cancer cells during hypoxia, leading to resistance to radio-chemotherapy [73–75]. However, treatment of G47Δ reduced the increase of CD133 + cells under hypoxic conditions. Moreover, G47Δ efficiently decreased the GSCs population under low oxygen levels, which could be useful to prevent the recurrence of GBM [76]. On the other hand, Friedman et al. demonstrated that there was no difference in resistance to oHSV-1 (ICP34.5 deleted) between CD133 + and CD133-GSCs. Moreover, their results showed that despite the increased expression of CD111 (nectine-1) under hypoxic conditions, the replication and efficacy of oHSV-1 in glioblastoma cells diminished [77].

### Adenovirus (Ad)

Adenovirus was the first OV that was approved by a national regulatory authority (China Food and Drug Administration (CFDA)) in 2005 [7].

In 2002, Alcoceba et al. modified adenovirus type 5 to be responsive to hypoxia. They hypothesized that deleting E1A and E1B could decrease the virus’ performance; therefore, they placed E1A under the control of an HRE-containing promoter. Their results showed that the cytotoxicity of oncolytic adenovirus increased during hypoxia. In addition, this virus could form multiple sites in which the viral load was high and cause damage in the hypoxic areas of solid tumors [78]. HYPR-Ad, as another oncolytic adenovirus that was developed through putting E1A under the control of a hypoxia/HIF-regulated promoter, causes cancer cell lysis under hypoxic conditions; they anticipated that HYPR-Ad could also kill cancer cells with an active hypoxia-inducible factor (HIF) during normoxia [79].

Malignant gliomas are characterized by the high degree of hypoxia and are extremely resistant to apoptosis and chemo-radiotherapy. In order to increase the antitumor activity of oncolytic adenovirus under hypoxic conditions, Hashimoto and colleagues used a human telomerase transcriptase promoter to drive E1 expression (OBP-301: Telomysin); in fact, they investigated the cytotoxicity of OBP-301 and Ad5 (wild type adenovirus) during normoxia and hypoxia. The results indicated that under hypoxic conditions, OBP-301 showed superior cytopathic activity and replication than Ad5 [80]. Likewise, Oh et al. designed a human telomerase reverse transcriptase (H5CmTERT) promoter to exploit hypoxic conditions to increase the responsiveness of oncolytic adenovirus (H5CmTERT-Ad). Moreover, they generated H5CmTERT-Ad expressing secretable tumor necrosis factor-related apoptosis-inducing ligand (H5CmTERT-Ad/TRAIL). According to their findings, H5CmTERT-Ad and H5CmTERT-Ad/TRAIL have greater cell killing capability in hypoxia than in normoxia. In the xenograft model, H5CmTERT-Ad/TRAIL could induce apoptosis, and hence, showed more anti-tumor efficacy and better distribution in tumor tissues than H5CmTERT-Ad [81]. In another study, Kwon et al. utilized a modified promoter to empower the oncolytic adenovirus during hypoxia. They used a modified human α-fetoprotein (hAFP) promoter with 6 or 12 copies of hypoxia response element (HRE) to regulate E1A expression of the oncolytic Ads known as Ad-HRE6/hAFP Δ19 and Ad-HRE12/hAFP Δ19. The results showed that Ad-HRE12/hAFP Δ19 exerted higher cytotoxicity and tumor selectivity than HRE6/hAFP Δ19 in hepatocellular carcinoma during hypoxia [82].

### Newcastle disease virus (NDV)

It has been shown that von Hippel-Lindau tumor suppressor protein (pVHL) is responsible for the degradation of HIF-1α during normoxia [83, 84]. Ch’ng and colleagues investigated the cancer cell killing capability of NDV (AF2240) in renal cell carcinoma (RCC) containing wild-type or deficient VHL under hypoxic conditions. Their results showed that NDV-infected RCC cells produced only IFN-β and not IFN-a, which was correlated with augmented STAT1 phosphorylation. In fact, NDV restored wild-type expression of VHL, which subsequently enhanced IFN-β expression, leading to increased STAT1 phosphorylation and cell death. They concluded that oncolytic NDV can potentially kill cancer cells during hypoxia [85].

### Vaccinia virus (VACV)

Hiley et al. investigated the antitumor activity of oncolytic vaccinia virus in a panel of pancreatic cancer cell lines (PDAC) under oxygen tension. They reported no differences in the synthesis of viral protein during normoxia or hypoxia, and there was a high viral titer in both conditions. Cytotoxicity of the virus was comparable among the cell lines except for CFPac1 and MiaPaca2 cells, in which the virus showed higher cytotoxicity during hypoxia [86].

### Vesicular stomatitis virus (VSV)

VSV is another OV that its entry into the cells is receptor-independent, and hence it can infect a wide variety of cell lines. In addition, it replicates in the cytoplasm without risk of host cell transformation, and the lack of anti-viral immunity in the general population makes it a promising candidate for eradicating cancer cells [87]. Connor et al. showed that during hypoxia, VSV could replicate more efficiently by producing a large amount of mRNA in comparison to normoxia. They found that VSV could dephosphorylate the eLF-4E translation factor, causing host translation inhibition in both conditions. Intratumoral or intravenous administration of the VSV in nude mice showed that the virus could infect and destroy the hypoxic regions of tumors [88]. Similarly, Zhou et al. indicated that VSV could efficiently kill cancer cells during hypoxia. They explained that VSV replication is dependent on glycolysis and glutamine metabolism. As a result, VSV could naturally target cancer cells in ascites that are usually exposed to hypoxic microenvironments. In addition, malignant ascites infected with oncolytic VSV showed enhancement of viral replication that could stem from glycolysis and glutamine metabolism augmentation in hypoxic areas of ascites [89].

### Reovirus (Rv)

RV, like VSV, can infect hypoxic cancer cells inherently and replicate preferentially in cancer cells with an active RAS pathway [90]. In 2010, Cho and colleagues demonstrated that reovirus could inhibit HIF-1 expression, but not at the transcriptional level, in HCT116 cells during hypoxia or CoCl2 treatment (hypoxia mimic). Interestingly, they showed that reovirus could reduce HIF-1 in VHL-/- renal carcinoma A498 and P53-/- HC116 cells, suggesting that decreases in HIF-1 levels were independent of VHL or P53 proteins. Moreover, it was found that infected VHL-/-A498 cells with increased HIF-1 expression were resistant to apoptosis compared to VHL + / + cells, but by utilizing YC-1 (HIF-1 inhibitor), apoptosis levels increased in VHL-negative A498 cells. Therefore, they suggested that oncolytic virotherapy combined with YC-1 could eradicate chemo-radio-resistant cancer cells with a high amount of HIF-1 expression (91). Likewise, Saraf et al. showed that prostate cancer cells (PCa) infected with oncolytic RV had decreased HIF-1 expression levels due to degradation and translational inhibition. They found that RV could replicate in and lyse PCa under hypoxic environments and induce apoptosis in cancer cells [92].

Aligned with prior research, Hotani et al. demonstrated that HIF-1 expression decreased following RV infection. They also studied the important question of whether the HIF-1 target gene will be downregulated following RV infection. They came to the conclusion that at 120 h post-systemic administration of RV, HIF-1 and its target genes were downregulated. In addition, they inactivated RV by UV and observed that the HIF-1 protein level was not altered, proposing that HIF-1 downregulation was dependent on RV replication. Moreover, no apoptosis was found at this time point, suggesting that RV-mediated killing of tumor cells might be independent of HIF-1 and its target gene’s protein levels. Besides, they investigated the replication of RV in the hypoxic region of tumors, and interestingly, they found the RV capsid protein in the hypoxic region of tumors 120 h after systemic administration of RV [93].

In another study, Figova and associates examined the effect of RV on brain tumor cells (U373) during hypoxia in vitro. Based on their results, RV could induce a cytopathic effect and subsequently kill cancer cells by a caspase-independent pathway. They also found that the cancer cells’ death caused by RV was not due to autophagy [94].

## Conclusion

Some OVs are naturally oncotropic and can eradicate hypoxic cancer cells inherently, including NDV, VSV, RV, and VACV. It seems that among the natural OVs, the vaccinia virus can replicate efficiently in either hypoxic or normoxic conditions. Although some OVs are inherently oncophilic, others must be modified to selectively target and kill cancer cells, such as HSV-1 and Ads. oHSVs have shown higher cytotoxicity under hypoxic conditions than oncolytic Ads. It appears that utilizing hypoxia-inducible promoters is an efficient way to compensate for the lower replication of Ads during hypoxia. Based on extensive studies, it seems that each OV reacts differently to the hypoxic microenvironments. But there are some concerns that should be considered before drawing a conclusion. First, there is no gold standard for measuring tumor hypoxia, which may differ between studies. Furthermore, oxygen tension in vitro experiments might be different from tissue oxygen microenvironments. Second, mimicking the hypoxic conditions in vitro might have distorted the results and affected the outcome of oncolytic virotherapy among different laboratories. Therefore, to deal with these problems and improve the treatment planning, a better understanding of TME, especially hypoxic conditions and its interaction with OVs, is indispensable. In addition, OVs as a single agent are not enough to eradicate all cancer cells due to the heterogeneity of cancer tissues. As a result, multiple approaches, including combination therapy, appear promising for creating a microenvironment in which OVs can diffuse and replicate more efficiently within hypoxic tumor cells.

## Data Availability

https://clinicaltrials.gov/.
